# Cost-Effectiveness of Endovascular vs Open Surgery for Chronic Limb-Threatening Ischemia

**DOI:** 10.1001/jamanetworkopen.2026.27335

**Published:** 2026-08-04

**Authors:** Zafar Zafari, Mehdi Najafzadeh, Mufaddal Mahesri, HoJin Shin, Philip P. Goodney, Michael S. Conte, Mark A. Creager, Michael D. Dake, John A. Kaufman, Richard J. Powell, Chris J. White, Michael B. Strong, Kenneth Rosenfield, Alik Farber, Matthew T. Menard, Niteesh K. Choudhry

**Affiliations:** 1 University of Maryland Institute for Health Computing, Bethesda; 2 Department of Practice, Sciences, and Health Outcomes Research, University of Maryland School of Pharmacy, Baltimore; 3 Medidata Solutions, Boston, Massachusetts; 4 Division of Pharmacoepidemiology and Pharmacoeconomics, Department of Medicine, Brigham and Women’s Hospital, Harvard Medical School, Boston, Massachusetts; 5 Heart and Vascular Center, Dartmouth Hitchcock Medical Center, Geisel School of Medicine at Dartmouth, Lebanon, New Hampshire; 6 Division of Vascular and Endovascular Surgery, University of California, San Francisco; 7 Department of Medical Imaging, University of Arizona Health Sciences, Tucson; 8 Division of Interventional Radiology, Oregon Health & Science University, Portland; 9 Department of Cardiovascular Diseases, The Ochsner Clinical School, New Orleans, Louisiana; 10 Division of Vascular and Endovascular Surgery, Brigham and Women’s Hospital, Harvard Medical School, Boston, Massachusetts; 11 Section of Vascular Medicine and Intervention, Massachusetts General Hospital, Harvard Medical School, Boston; 12 Division of Vascular and Endovascular Surgery, Boston Medical Center, Boston University Chobanian & Avedisian School of Medicine, Boston, Massachusetts

## Abstract

**Importance:**

Choosing between endovascular and open (bypass) surgical revascularization for chronic limb-threatening ischemia has major implications for clinical practice.

**Objective:**

To evaluate the cost-effectiveness of endovascular vs bypass surgical revascularization.

**Design, Setting, and Participants:**

This economic evaluation used individual-level data from the Best Endovascular vs Best Surgical Therapy for Patients With Chronic Limb Ischemia (BEST-CLI) trial, with the first patient enrolled August 28, 2014, and the final October 18, 2019, for a median follow-up of 2.7 years. An individual-level, continuous-time Markov model with health states based on adjudicated clinical events from BEST-CLI was developed. Rates of clinical outcomes, health utilities, and health care resource use were derived from trial data. Unit costs came from Medicare insurance claims data and the physician fee schedule. The data were analyzed between June 27, 2025, and May 28, 2026.

**Main Outcomes and Measures:**

The main outcomes were incremental cost per life-years gained, incremental quality-adjusted life-years (QALYs) gained, incremental net monetary benefit, and cost per major events of revascularization and amputation avoided over a 5- and 10-year time horizon. One-way and probabilistic sensitivity analyses were performed to quantify uncertainty.

**Results:**

The cohort included 1434 patients from BEST-CLI (mean [SD] age, 67 [10] years; 1026 male [71.5%]). In the base case analysis, over a 5-year time horizon, the mean per-person direct medical costs were $118 559 (95% credible interval [CrI], $86 978-$157 152) for bypass surgery and $125 535 (95% CrI, $96 205-$160 357) for endovascular surgery. The mean survival per person was 3.84 years (95% CrI, 3.74-3.93 years) and 3.78 years (95% CrI, 3.61-3.94 years) for bypass and endovascular surgery, respectively. The mean QALYs per person were 2.53 (95% CrI, 2.34-2.70) for bypass surgery and 2.48 (95% CrI, 2.28-2.67) for endovascular surgery. Bypass surgery dominated endovascular surgery with respect to both costs per life-year and per QALY gained. The results over 10 years were consistent with those of the 5-year BEST-CLI follow-up. In the Monte Carlo simulation, there was a 93% chance that bypass surgery was more cost-effective than endovascular surgery.

**Conclusions and Relevance:**

This cost-effectiveness study found that bypass surgery was more cost-effective in most probabilistic simulations; however, uncertainty remained, highlighting the need for future research to identify subgroups in whom each approach may be cost-effective.

## Key Points

**Question:**

What is the cost-effectiveness of endovascular vs bypass surgical revascularization for chronic limb-threatening ischemia?

**Findings:**

In this economic evaluation in 1434 patients, bypass surgery was associated with fewer major reinterventions and amputations, greater life-years and quality-adjusted life-years gained, and lower costs compared with endovascular surgery. In probabilistic sensitivity analyses, bypass surgery had a 93% probability of being cost-effective at conventional willingness-to-pay thresholds.

**Meaning:**

These findings suggest that bypass may have superior outcomes at lower cost compared with endovascular surgical revascularization, although residual uncertainty remains, highlighting the need for further research to identify patient subgroups most likely to benefit from each strategy.

## Teaser

This economic evaluation assesses the cost-effectiveness and life-years gained of endovascular vs bypass revascularization for chronic limb-threatening ischemia.

## Introduction

Chronic limb-threatening ischemia (CLTI) is the most severe manifestation of atherosclerotic lower-extremity peripheral artery disease.^1^ Globally, more than 200 million people have peripheral artery disease,^2^ with CLTI affecting 11% of these individuals.^1^ Chronic limb-threatening ischemia is associated with a high risk of leg amputation and adverse cardiovascular outcomes, including myocardial infarction (MI), stroke, and cardiovascular death,^2,3^ and has substantial economic implications as it results in an estimated annual cost approximating $12 billion in the US.^3^

Along with medical therapy to reduce cardiovascular risk and limb care to control infection and optimize wound healing, treatment of CLTI requires revascularization to improve limb perfusion with either an endovascular or open surgical (bypass) approach.^4^ The clinical effectiveness of bypass compared with endovascular treatment strategies for CLTI was evaluated in the Best Endovascular vs Best Surgical Therapy for Patients With Critical Limb Ischemia (BEST-CLI) trial. This study found that among patients who were suitable candidates for surgical bypass using a single-segment great saphenous vein (SSGSV) (designated as cohort 1 in the trial), those randomized to bypass had a 33% risk reduction of the primary outcome of the composite of all-cause death or major adverse limb events (defined as above-ankle amputation or major limb reintervention) compared with those in the endovascular arm, as well as significant reductions in the secondary end points of above-ankle amputation and major reintervention.^5^ Health-related quality of life was significantly and clinically improved from baseline to all follow-up for both groups across all measures. While the difference between treatment groups was not clinically meaningful, patients in the endovascular arm had statistically significant greater improvements on some measures (eg, Vascular Quality of Life Questionnaire) but not others (eg, European Quality of Life 5-Dimension Questionnaire).^6^

Given differences in clinical outcomes observed in the clinical trial, we evaluated the cost-effectiveness of endovascular vs bypass using data from the BEST-CLI trial. We hypothesized that bypass would be more cost-effective because of lower rates of major adverse limb events and amputation observed in the BEST-CLI trial.

## Methods

This economic evaluation used individual-level data from the BEST-CLI trial. The BEST-CLI was a prospective, randomized, multicenter trial that compared the effectiveness of endovascular vs bypass revascularization in patients with CLTI due to infrainguinal peripheral artery disease.^5^ The trial enrolled the first participant on August 28, 2014, and the final participant on October 18, 2019. The primary institutional review board for the trial was at Brigham and Women’s Hospital, which is where the clinical coordinating center was based. The trial protocol was approved by the ethics committee or the national equivalent at each participating site.^7^ All the participants provided written informed consent. This study followed the Consolidated Health Economic Evaluation Reporting Standards (CHEERS) reporting guideline.^8^

### Statistical Analysis

The data analysis was performed between June 27, 2025, and May 28, 2026. We developed an individual-level continuous-time Markov model using the hesim package, version 0.5.8 in R, version 4.6.1 (R Foundation for Statistical Computing), a validated modular and computationally efficient R package for health economic simulation modeling and decision analysis.^9,10,11^ The model consisted of 5 health states representing the occurrence of the prespecified and adjudicated clinical events from the BEST-CLI trial. These states included (1) no event after index surgery; (2) post minor reintervention; (3) post major reintervention, indicating a major index limb reintervention such as a new bypass graft or jump or interposition graft revision; (4) post amputation, and (5) death. We also modeled nonfatal MI or stroke as recurrent events in the continuous-time Markov model, meaning that they could occur during follow-up in each of the health states other than death. Given the continuous-time nature of the model, patients remained in a given state until the next event occurred. The model schema is shown in eFigure 1 in Supplement 1.

#### Model Parameters

The model parameters, including transition probabilities, resource use estimates, and health-state utility values, were derived directly from cohort 1 of the BEST-CLI trial. Cohort 1 consisted of individuals with an adequate SSGSV and included 1434 of the 1830 patients enrolled in the trial. Table 1 shows the model input parameters for demographics, comorbidities, baseline medication use, health state utilities, and costs.^12,13,14,15,16,17,18^

**Table 1.** Model Input Parameters for Demographics, Comorbidities, Health State Utilities, and Costs

Parameter	Base case estimate, mean (SD)	Source
Demographics^a^		
Age, y	67 (10)	BEST-CLI trial data
Sex, No. (%)		
Female	408 (28.5)	
Male	1026 (71.5)	
BMI	28.5 (6.2)	
Comorbidities (prevalence)^b^		
Coronary artery disease	0.42 (0.49)	BEST-CLI trial data
Diabetes	0.73 (0.45)	
Chronic heart failure	0.06 (0.23)	
Chronic kidney disease	0.28 (0.45)	
COPD	0.15 (0.36)	
Hypertension	0.75 (0.44)	
Stroke	0.13 (0.34)	
Baseline medication use (prevalence)^b^		
Direct oral anticoagulant	0.04 (0.19)	BEST-CLI trial data
Use of warfarin	0.07 (0.25)	
Health state utilities, mean (SE)^c^		
No event (post index surgery)	0.664 (0.03)	BEST-CLI trial data
Post minor reintervention	0.664 (0.03)	
Post major reintervention	0.659 (0.07)	
Post amputation	0.548 (0.10)	
Index surgery costs, mean (SE), $^d^		
Bypass	14 273 (3568)	Costs calculated from Medicare claims using methodologies from Brahmandam et al^12^ and Goodney et al^13^
Endovascular	16 084 (4021)	
One-time costs, mean (SE), $^d^		
Minor reintervention	4600 (1150)	Costs corresponding to 1 y after the event of interest calculated as use data × unit costs
Major reintervention	25 600 (6400)	
Amputation	23 000 (5750)	
Nonfatal MI or stroke	28 600 (6000)	
Maintenance costs (mean annual), mean (SE), $^d^		
No event (post index surgery)	22 000 (5500)	Average annual maintenance costs >1 y after the event of interest calculated as use data × unit costs
Post minor reintervention	31 700 (7925)	
Post major reintervention	26 300 (6575)	
Post amputation	33 200 (8300)	

Abbreviations: BEST-CLI, Best Endovascular vs Best Surgical Therapy for Patients With Chronic Limb Ischemia; BMI, body mass index (calculated as weight in kilograms divided by height in meters squared); COPD, chronic obstructive pulmonary disease; MI, myocardial infarction.

^a^ For demographics, continuous variables were modeled using normal distributions, and binary variables were modeled using binomial distributions; BEST-CLI trial data were used for these distributions.

^b^ Presence of comorbidities and baseline medication use modeled using binomial distributions; BEST-CLI trial data were used for these distributions.

^c^ Utilities modeled using β distributions; BEST-CLI trial data were used for these distributions. No-event and minor reintervention states were assigned the same utility because observed European Quality of Life 5-Dimension Questionnaire score differences were not statistically or clinically meaningful.

^d^ Index surgery, one-time, and maintenance costs were modeled using γ distributions; BEST-CLI trial data were used for use, and publicly available data and published literature were used for unit costs.^14,15,16,17,18^

#### Transition Probabilities

We used a progressive, multistate, cause-specific hazards models to estimate transition-specific hazards for minor reintervention, major reintervention, above-ankle amputation, and death using patient-level BEST-CLI data. Individuals could transition between clinically defined health states over time, with transition-specific risks estimated conditional on the patient’s current state. For each transition, competing transitions from the same state were modeled using cause-specific hazards models. Individuals experiencing a competing event subsequently contributed person-time to the new state occupied following that event. The following covariates from the BEST-CLI trial were included in the models: treatment arm (ie, bypass vs endovascular), age, sex, body mass index (BMI; calculated as weight in kilograms divided by height in meters squared), hypertension, diabetes, prior stroke, coronary artery disease, congestive heart failure, chronic kidney disease, chronic obstructive pulmonary disease, use of a direct oral anticoagulant, and use of warfarin. These covariates were selected using least absolute shrinkage and selection operator regressions along with clinical judgment. Race and ethnicity were not included in the final models after consideration of the least absolute shrinkage and selection operator results and clinical judgment to maintain a parsimonious model. We validated these models using the rms package in R. The exponent of the hazard ratio represented the time to event for each outcome for individual patients.

#### Direct Medical Costs

We used health care resource use data from the BEST-CLI trial to estimate the costs. Our analysis was conducted from the 2024 US payer perspective. For each health state in the model in a given treatment arm, we estimated the unit costs for the number of annualized hospitalization days^14^; intensive care unit days^15^; emergency department visits^16^; dialysis days^17^; tracheostomy, gastrointestinal endoscopy, and cardiac catheterization procedures; computed tomography scans; magnetic resonance imaging scans; ultrasonography examinations; echocardiography examinations; cardiac stress tests; physical therapy sessions; outpatient visits; outpatient rehabilitation hours; and inpatient rehabilitation hours.^18^ We then multiplied estimates of these resources by their respective unit costs, derived from publicly available data sources,^14,15,16,17,18^ to estimate the average direct medical costs associated with each health state in each treatment arm. The costs of index procedures were based an analysis of Centers for Medicare & Medicaid Services claims data representing costs for the procedure and the next 30 days.

For hospitalization costs of nonfatal MI or stroke, we used the average costs of acute MI and cerebrovascular event hospitalizations informed by nationally representative US estimates from the National Inpatient Sample^19^ and inflated to 2025 US dollars using the health care component of the Personal Consumption Expenditure Index.^20^

#### Health State Utility Values

We used European Quality of Life 5-Dimension Questionnaire scores,^21,22^ a self-reported general health-related quality-of-life index, collected during the BEST-CLI trial to calculate the mean (SE) of utility values associated with each of 4 model health states in each treatment arm. The model input parameters for annual transition rates, one-time and maintenance costs, and health state utilities are reported in Table 2.

**Table 2.** Model Input Parameters for Mean Annual Transition Rates

Annual transition rates^b^	Base case estimate, mean (SD)^a^	
	Bypass surgery	Endovascular surgery
No event to minor reintervention	0.077 (0.019)	0.086 (0.021)
No event to major reintervention	0.023 (0.006)	0.066 (0.017)
No event to amputation	0.025 (0.016)	0.035 (0.023)
No event to death	0.064 (0.062)	0.070 (0.068)
Minor reintervention to major reintervention	0.063 (0.115)	0.125 (0.227)
Minor reintervention to amputation	0.075 (0.070)	0.054 (0.050)
Minor reintervention to death	0.282 (0.256)	0.159 (0.145)
Major reintervention to amputation	0.056 (0.068)	0.065 (0.079)
Major reintervention to death	0.189 (0.171)	0.206 (0.185)
Amputation to death	0.240 (0.178)	0.262 (0.194)
Rate of nonfatal MI or stroke, mean (SE)^c^	0.064 (0.005)	0.069 (0.005)

Abbreviation: MI, myocardial infarction.

^a^ The standard deviation represents the transition rates across individuals in the model.

^b^ Annual transition rates were modeled using log-normal distributions in probabilistic sensitivity analyses. In the continuous-time Markov model, exponentially distributed event times were generated using these transition rates. BEST-CLI trial data were used.

^c^ The standard error represents that of the annual rate of nonfatal MI or stroke in the probabilistic sensitivity analyses, which was modeled as an event.

#### Base Case Analysis

For the base case, we conducted our analyses over time horizons of 5 and 10 years. All future costs and quality-adjusted life-year (QALY) gains were discounted at 3%, consistent with recommendations of the US Second Panel on Cost-Effectiveness in Health and Medicine.^23^ We calculated the cost-effectiveness of endovascular vs bypass surgery in terms of both their incremental costs per life-year gained and incremental costs per QALY gained.

We also estimated the incremental net monetary benefit (INMB) of endovascular vs bypass surgery, which was calculated by multiplying the incremental QALYs gained by a willingness-to-pay value of $100 000 per QALY and then subtracting the incremental costs. Additionally, we calculated the incremental costs to avoid the individual health states in the model, ie, major intervention, above-ankle amputation, and nonfatal MI or stroke.

#### Sensitivity Analysis

We performed extensive sensitivity analyses based on assumed probability distributions for the model input parameters. We conducted 1-way sensitivity analyses on each of the input parameters, including patient characteristics (ie, age, sex, BMI, and presence of major baseline comorbidities), transition probabilities, health state utility values, and medical costs. We summarized these results using a tornado diagram.

We conducted probabilistic analyses using a Monte Carlo simulation with 10 000 iterations. Model input parameters were randomly drawn from their probability distributions in each iteration, and the outcomes were estimated for each set of input parameters. We presented the results of the probabilistic analyses using 95% credibility intervals (95% CrIs) covering 95% of the random simulations. In addition, we plotted the results of the probabilistic analyses in a cost-effectiveness plane and a cost-effectiveness acceptability curve.

#### Subgroup Analyses

We performed subgroup analyses based on the location of revascularization (ie, femoropopliteal, infrapopliteal or tibial) using published BEST-CLI subgroup hazard ratios.^24,25^ Because location-specific procedural cost data were not available, we held intervention costs equal to the base case analysis.

We also evaluated the impact of different endovascular procedures, including atherectomy and antiproliferative drug technology use. In these analyses, we applied the technology-specific hazard ratios from published BEST-CLI subgroup analyses to the base case endovascular transition hazards. Because detailed technology-specific procedural cost data were unavailable, and given substantial variability in reimbursement and procedural costs across practice settings, we varied incremental technology costs across clinically plausible ranges added to the base case endovascular index procedure cost, specifically $0 to $3000 for antiproliferative drug technology^26^ and $0 to $10 000 for atherectomy.^27^ These analyses were intended to explore whether plausible variation in endovascular technology effectiveness and cost could materially alter cost-effectiveness conclusions.

## Results

The BEST-CLI’s cohort 1 included 1434 patients with SSGSV (mean [SD] age, 67 [10] years; 408 female [28.5%] and 1026 male [71.5%]). Patients were followed up for up to 7 years (median [IQR], 2.7 [1.6-4.1] years). Their mean (SD) BMI was 28.5 (6.2).

### Model Calibration

Our simulation model closely replicated the observed event rates from the BEST-CLI trial over the 5-year follow-up (eFigure 2 in Supplement 1). Similarly, the observed relative risks of the 4 clinical outcomes in the trial fell within the 95% CrIs of the simulation model’s relative risks (eFigure 3 in Supplement 1).

### Base Case Analyses

Results for our base case analyses are shown in Table 3. For a 5-year time horizon, patients treated with bypass surgery were estimated to have mean direct medical costs of $118 559 (95% CrI, $86 978-$157 152) and a mean survival of 3.84 years (95% CrI, 3.74-3.93 years). Patients treated with endovascular surgery had mean direct medical costs of $125 535 (95% CrI, $96 205-$160 357) and a mean survival of 3.78 years (95% CrI, 3.61-3.94 years). As a consequence, endovascular surgery was dominated by bypass surgery (ie, bypass surgery was more effective and less costly than endovascular surgery) in terms of the incremental costs per life-year gained.

**Table 3.** Main Results for the Cost-Effectiveness of Endovascular vs Bypass Surgery for Chronic Limb-Threatening Ischemia

Variable	Estimate, mean (95% CrI), $			Incremental cost per life-year gained	Incremental cost per QALY	INMB, mean (95% CrI), $^a^
	Bypass surgery	Endovascular surgery	Difference (endovascular − bypass)			
**5-y Time horizon**						
Cost	118 559 (86 978 to 157 152)	125 535 (96 205 to 160 357)	6976 (−6585 to 20 438)	Dominated	Dominated	−11 841 (−28 081 to 3711)
Survival	3.84 (3.74 to 3.93)	3.78 (3.61 to 3.94)	−0.06 (−0.25 to 0.13)			
QALY	2.53 (2.34 to 2.70)	2.48 (2.28 to 2.67)	−0.05 (−0.18 to 0.08)			
**10-y Time horizon**						
Cost	179 074 (134 547 to 232 757)	186 598 (145 023 to 234 422)	7523 (−14 052 to 28 854)	Dominated	Dominated	−20 321 (−47 272 to 5726)
Survival	5.91 (5.71 to 6.11)	5.74 (5.32 to 6.17)	−0.16 (−0.64 to 0.30)			
QALY	3.86 (3.57 to 4.15)	3.74 (3.36 to 4.12)	−0.13 (−0.45 to 0.19)			

Abbreviations: CrI, credible interval; INMB, incremental net monetary benefit; QALY, quality-adjusted life year.

^a^ Calculated from multiplying the willingness-to-pay value of $100 000 per QALY by incremental QALYs gained minus the incremental costs.

The mean QALYs per person were 2.53 (95% CrI, 2.34-2.70) and 2.48 (95% CrI, 2.28-2.67) for bypass and endovascular surgery, respectively, resulting in a dominated incremental cost per QALY gained and a mean INMB of −$11 841 (95% CrI, −$28 081 to $3711) for endovascular vs bypass surgery.

The findings for the 10-year time horizon were similar to the 5-year findings. In the base case analysis, endovascular surgery was dominated by bypass surgery with respect to both incremental costs per life-year gained and incremental costs per QALY gained. The INMB for endovascular vs bypass was −$20 321 (95% CrI, −$47 272 to $5726).

Results in terms of major events are presented in the eTable in Supplement 1. Over a 5-year time horizon, bypass surgery reduced spending by $52 724 (95% CrI, −$170 056 to $55 731) for every major reintervention avoided and reduced spending by $197 066 (95% CrI, −$1 903 460 to $1 405 687) for every amputation avoided. Results over a 10-year time horizon were similar.

### Sensitivity Analyses

**Figure 1.**
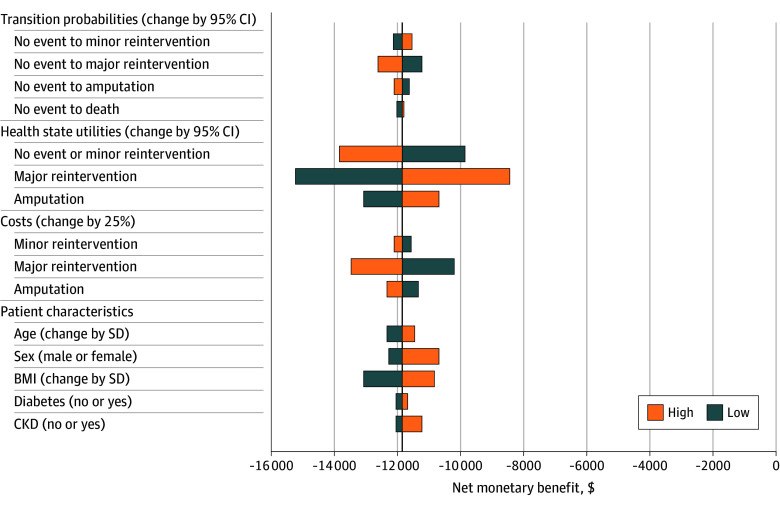
Tornado Diagram of One-Way Sensitivity Analysis of Incremental Net Monetary Benefit Shown is the association of varying key model input parameters with the incremental net monetary benefit of endovascular vs bypass surgery for chronic limb-threatening ischemia. BMI indicates body mass index; CKD, chronic kidney disease.

Figure 1 shows the results of the 1-way sensitivity analysis over a 5-year time horizon. Changing the health state utility values of the no-event and post–major reintervention states by their corresponding 95% CIs (no event, 0.61-0.71; post major reintervention, 0.52-0.79) changed the INMB from a low of −$13 855 to a high of −$9852, and from −$15 225 to −$8449, respectively. Changing the BMI and age by their SDs changed the INMB from −$13 087 to −$10 827 and from −$12 636 to −$11 460, respectively. Varying the cost of reintervention by 25% changed the INMB from −$13 471 to −$10 211.

**Figure 2.**
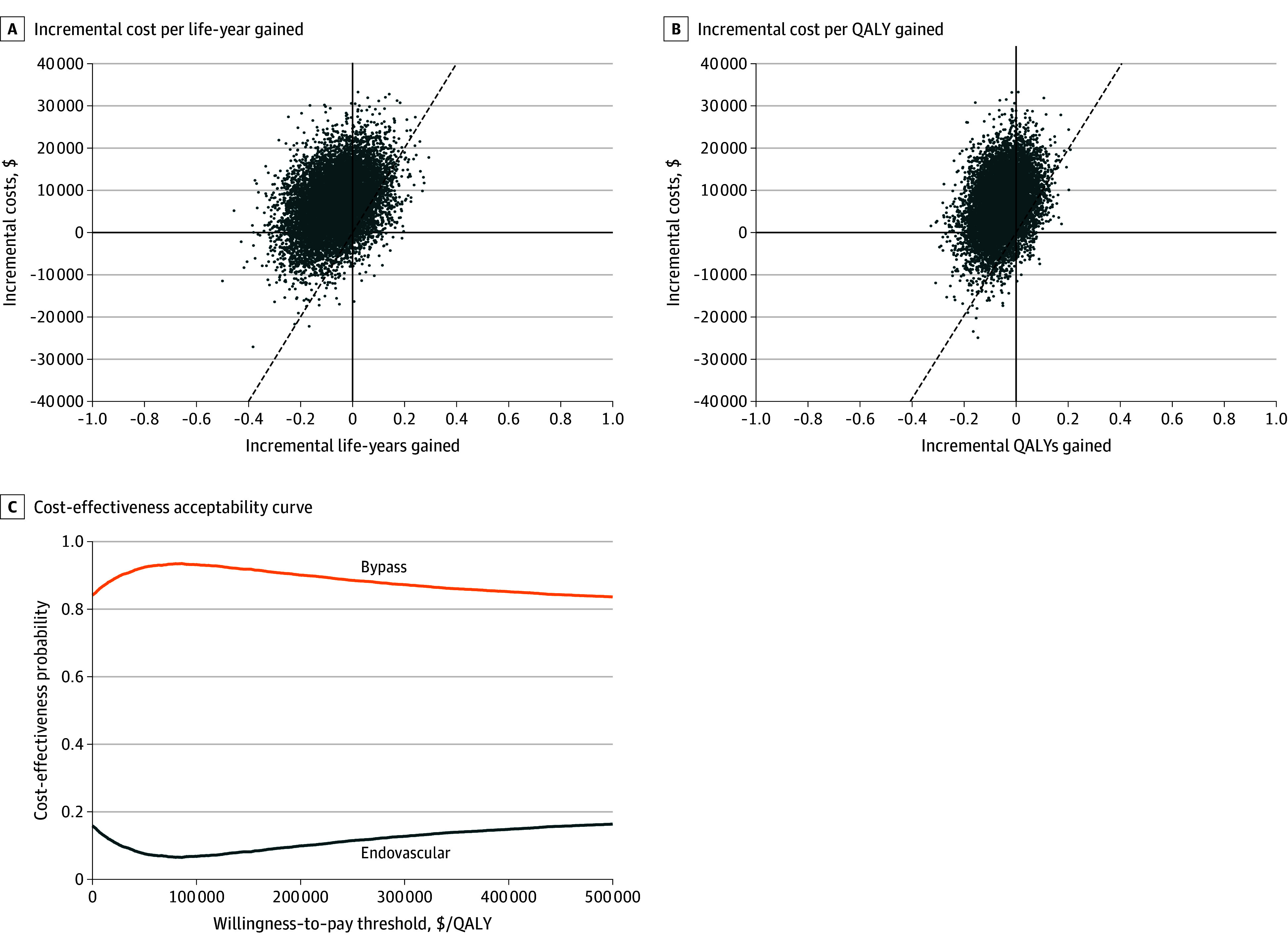
Scatterplots and Line Graphs of Probabilistic Cost-Effectiveness Results for Endovascular vs Bypass Surgery for Chronic Limb-Threatening Ischemia QALY indicates quality-adjusted life-year.

Figure 2 shows the results of the probabilistic analysis. At a willingness-to-pay threshold of $100 000 per QALY gained, bypass surgery was more cost-effective than endovascular surgery in 93% of the Monte Carlo simulations. The probability of cost-effectiveness of bypass surgery reduced to 90% and 82% at willingness-to-pay thresholds of $150 000 per QALY gained and $500 000 per QALY gained, respectively.

### Subgroup Analyses

For patients undergoing femoropopliteal revascularization, endovascular surgery was dominated by bypass surgery when considering the point estimates of the incremental costs per QALY gained (INMB, −$11 047; 95% CrI, −$27 135 to $3247) compared with bypass surgery over 5 years. Similarly, for the infrapopliteal or tibial revascularization subgroup, endovascular surgery was dominated by bypass surgery based on the point estimates of the incremental costs per QALY gained with a 5-year INMB of −$9968 (95% CrI, −$25 665 to $4311) compared with endovascular surgery. Exploratory subgroup analyses based on the type of endovascular procedure performed found that patients undergoing atherectomy had an INMB of −$14 543 (95% CrI, −$32 906 to $2736) compared with bypass surgery over a 5-year period under the assumption of no added procedural cost. Assuming that atherectomy costs $5000 or $10 000 led to an INMB of −$19 543 (95% CrI, −$37 906 to −$2264) and −$24 543 (95% CrI, −$42 906 to −$7264), respectively. For an endovascular procedure with antiproliferative drug technology, the INMB for endovascular surgery plus drug vs bypass surgery over 5 years was −$2735 (95% CrI, −$17 359 to $10 803), −$4235 (95% CrI, −$18 859 to $9303), and −$5735 (95% CrI, −$20 359 to $7803) assuming no added intervention cost, added intervention costs of $1500, and added intervention costs of $3000, respectively (eFigure 4 in Supplement 1).

## Discussion

This economic evaluation assessed the cost-effectiveness of endovascular compared with bypass surgery for patients with CLTI by conducting an individual-level simulation model using data from the BEST-CLI trial. When considering the point estimates of incremental costs per each additional year of survival and incremental costs per QALY gained, bypass surgery was associated with lower costs and better survival and QALYs over both a 5- and 10-year time horizon in our base case analysis, meaning that bypass surgery was dominant compared with endovascular surgery. Similarly, every major clinical event avoided by bypass surgery was associated with large cost savings.

Bypass was also cost-effective in the majority of the probabilistic sensitivity analysis simulations. Nevertheless, the remaining uncertainty suggests that future research is needed to identify subgroups for whom each of these interventions is more definitively cost-effective. Furthermore, additional value of information may be gained from better characterizing the relative effects and costs of these 2 revascularization approaches for CLTI.^28^

Only 2 prior randomized clinical trials have evaluated the cost-effectiveness of open and endovascular revascularization approaches for patients with CLTI.^29^ Similar to our findings, a cost-utility analysis of the Bypass vs Angioplasty in Severe Ischemia of the Leg (BASIL) trial^30^ found that bypass surgery was associated with greater health-related–adjusted survival, although unlike our findings, the trial showed a slightly higher spending for bypass surgery, with a resultant incremental cost per QALY gained of $184 492.^31^ A cost-utility analysis of the more recently completed BASIL 2 trial found that bypass surgery was associated with substantially higher costs and slightly higher QALYs than endovascular surgery, with a resultant incremental cost per QALY gained for bypass compared with endovascular surgery of $730 000.^32^ Similarly, these studies found substantial uncertainty in the results of their base case analyses, and the cost-effectiveness analysis of BASIL 2 found substantial cost savings per amputation avoided.

There are numerous challenges comparing the results of these prior trials with those from BEST-CLI. Both trials were conducted in the UK. The BASIL trial was completed more than 20 years ago, during which time endovascular treatment was mostly done using balloon angioplasty, quality-of-life data were missing for approximately one-third of patients, and the analysis only considered in-hospital costs. The BASIL 2 trial participants were older than those in BEST-CLI, and all had infrapopliteal revascularization; in contrast, 42% of BEST-CLI patients had femoropopliteal procedures. Both BASIL cost-effectiveness analyses considered shorter time horizons than we evaluated and were conducted by taking cohort-level data observed during these trials. In contrast, we built our Markov model at the individual level, which enabled quantifying outcomes for different patient subgroups. We looked at a wide set of covariates that are associated with the clinical outcomes, including reinterventions, amputation, and death, and chose covariates based on both their clinical relevance and statistical performance.

### Limitations

It is important to acknowledge some of the limitations of our approach. First, while our base case estimates were based on current procedural reimbursements from Medicare claims for endovascular and open surgical procedures, regional and year-to-year variations in Centers for Medicare & Medicaid Services payments, as well as whether the procedure was performed in inpatient or outpatient settings, could potentially affect the direction and magnitude of the associations seen in our analysis.^33,34,35^ To limit the impact of these factors, we based cost estimates for open and endovascular treatments on Medicare diagnosis-related group payments made to hospitals for these services.^34^ Differences in the characteristics and extent of the procedure may affect hospital charges,^36^ but these differences are muted with the diagnosis-related group payment.^37^ Second, the rate of initial endovascular technical failure in BEST-CLI was relatively high compared with some contemporary endovascular studies.^5^ Although technical failure was not explicitly modeled as a separate transition state because sufficiently granular procedural-level data were unavailable, the current intention-to-treat analysis inherently incorporated downstream consequences of technical failure within the endovascular arm through observed differences in reintervention, amputation, mortality, and health care use outcomes. Finally, although we performed exploratory subgroup analyses according to revascularization location (femoropopliteal and infrapopliteal or tibial revascularization) and the use of specific endovascular technologies (ie, atherectomy, antiproliferative drug technology use), the cost-effectiveness of endovascular therapy may still depend on operator technical skill, patient selection, lesion complexity, and procedural- or device-specific factors not fully captured within the present model.

## Conclusions

This economic evaluation of the cost-effectiveness of endovascular vs bypass surgery found greater survival, lower rates of major adverse limb events, and greater health-related quality of life at a lower cost for bypass surgery in the base case analysis and the majority of probabilistic sensitivity analysis simulations. The residual uncertainty in our estimates justifies future research to identify subgroups for whom each of these approaches may definitively be cost-effective.

## Notes

Supplement 1. **eTable.** Results of Cost-Effectiveness Analysis Presented as Incremental Costs per Clinical Event Avoided **eFigure 1. **Schematic Representation of the Cost-Effectiveness Model **eFigure 2. **Calibration Plots for Trial-Observed Versus Regression-Estimated Probabilities of the Clinical Outcomes (Reintervention, Amputation, MACE, and Death) Within 365 Days **eFigure 3. **Trial-Observed Versus Model-Estimated Relative Risks of the Clinical Outcomes (Reintervention, Amputation, Nonfatal MI or Stroke, and Death) for Bypass Versus Endovascular Surgery **eFigure 4. **Incremental Net Monetary Benefit of Endovascular Procedures With Atherectomy or Antiproliferative Drug Technology Use Versus Bypass

Supplement 2.
Data Sharing Statement

## References

[1] Conte MS, Bradbury AW, Kolh P, White JV, Dick F, Fitridge R, ; GVG Writing Group. Global vascular guidelines on the management of chronic limb-threatening ischemia. J Vasc Surg. 2019;69(6S):3S-125S.e40. doi:10.1016/j.jvs.2019.02.016 31159978 PMC8365864

[2] Fowkes FGR, Rudan D, Rudan I, . Comparison of global estimates of prevalence and risk factors for peripheral artery disease in 2000 and 2010: a systematic review and analysis. Lancet. 2013;382(9901):1329-1340. doi:10.1016/S0140-6736(13)61249-0 23915883

[3] Mustapha JA, Katzen BT, Neville RF, . Determinants of long-term outcomes and costs in the management of critical limb ischemia: a population-based cohort study. J Am Heart Assoc. 2018;7(16):e009724. doi:10.1161/JAHA.118.009724 30369325 PMC6201392

[4] Farber A. Chronic limb-threatening ischemia. N Engl J Med. 2018;379(2):171-180. doi:10.1056/NEJMcp1709326 29996085

[5] Farber A, Menard MT, Conte MS, ; BEST-CLI Investigators. Surgery or endovascular therapy for chronic limb-threatening ischemia. N Engl J Med. 2022;387(25):2305-2316. doi:10.1056/NEJMoa2207899 36342173

[6] Menard MT, Farber A, Powell RJ, ; BEST-CLI Investigators. Quality of life in patients with chronic limb-threatening ischemia treated with revascularization. Circulation. 2024;149(16):1241-1253. doi:10.1161/CIRCULATIONAHA.123.065277 38597097 PMC11360227

[7] Best endovascular vs best surgical therapy in patients with critical limb ischemia (BEST-CLI). ClinicalTrials.gov identifier: NCT02060630. Updated April 11, 2023. Accessed September 21, 2025. https://www.clinicaltrials.gov/ct2/show/NCT02060630

[8] Husereau D, Drummond M, Augustovski F, ; CHEERS 2022 ISPOR Good Research Practices Task Force. Consolidated Health Economic Evaluation Reporting Standards 2022 (CHEERS 2022) statement: updated reporting guidance for health economic evaluations. Value Health. 2022;25(1):3-9. doi:10.1016/j.jval.2021.11.1351 35031096

[9] Incerti D, Jansen JP, Clements M; R Core Team. hesim: Health economic simulation modeling and decision analysis. The Comprehensive R Network. 2024. Accessed March 15, 2025. https://cran.r-project.org/web/packages/hesim/index.html

[10] Incerti D, Jansen JP. hesim: Health economic simulation modeling and decision analysis. arXiv. Preprint posted online February 18, 2021. https://arxiv.org/abs/2102.09437

[11] Jansen JP, Incerti D, Linthicum MT. Developing open-source models for the US health system: practical experiences and challenges to date with the Open-Source Value Project. Pharmacoeconomics. 2019;37(11):1313-1320. doi:10.1007/s40273-019-00827-z 31392665 PMC6860458

[12] Brahmandam A, Zhen X, Mao J, . Outcomes and costs of lower extremity peripheral vascular interventions with intravascular ultrasound. J Vasc Surg. 2023;77(6):e184-e185. doi:10.1016/j.jvs.2023.03.253

[13] Goodney PP, Travis LL, Brooke BS, . Relationship between regional spending on vascular care and amputation rate. JAMA Surg. 2014;149(1):34-42. doi:10.1001/jamasurg.2013.4277 24258010 PMC4279246

[14] Hospital expenses per adjusted inpatient day. Kaiser Family Foundation. Accessed July 29, 2024. https://www.kff.org/health-costs/state-indicator/expenses-per-inpatient-day/

[15] Dasta JF, McLaughlin TP, Mody SH, Piech CT. Daily cost of an intensive care unit day: the contribution of mechanical ventilation. Crit Care Med. 2005;33(6):1266-1271. doi:10.1097/01.CCM.0000164543.14619.00 15942342

[16] Mirel LB, Carper K. Trends in health care expenditures for the elderly, age 65 and older: 2001, 2006, and 2011. In: *Statistical Brief (Medical Expenditure Panel Survey (US))*. Agency for Healthcare Research and Quality; 2001. Accessed June 14, 2025. https://www.ncbi.nlm.nih.gov/books/NBK476262/29360330

[17] Trish E, Fiedler M, Ning N, Gascue L, Adler L, Lin E. Payment for dialysis services in the individual market. JAMA Intern Med. 2021;181(5):698-699. doi:10.1001/jamainternmed.2020.7372 33749739 PMC7985814

[18] PFS look-up tool. Centers for Medicare & Medicaid Services. Accessed July 29, 2024. https://www.cms.gov/medicare/physician-fee-schedule/search/overview

[19] Tajeu GS, Ruiz-Negrón N, Moran AE, . Cost of cardiovascular disease event and cardiovascular disease treatment-related complication hospitalizations in the United States. Circ Cardiovasc Qual Outcomes. 2024;17(3):e009999. doi:10.1161/CIRCOUTCOMES.123.009999 38328916 PMC11099996

[20] Table 2.4.4.U. price indexes for personal consumption expenditures by type of product. Bureau of Economic Analysis. Accessed May 24, 2026. https://apps.bea.gov/iTable/?reqid=19&step=2&isuri=1&categories=underlying#eyJhcHBpZCI6MTksInN0ZXBzIjpbMSwyLDNdLCJkYXRhIjpbWyJjYXRlZ29yaWVzIiwiU3VydmV5Il0sWyJOSVBBX1RhYmxlX0xpc3QiLCIyMDE2Il1dfQ==

[21] EQ-5D-5L. EuroQol. Accessed March 17, 2025. https://euroqol.org/information-and-support/euroqol-instruments/eq-5d-5l/

[22] Rabin R, de Charro F. EQ-5D: a measure of health status from the EuroQol Group. Ann Med. 2001;33(5):337-343. doi:10.3109/07853890109002087 11491192

[23] Sanders GD, Neumann PJ, Basu A, . Recommendations for conduct, methodological practices, and reporting of cost-effectiveness analyses: second panel on cost-effectiveness in health and medicine. JAMA. 2016;316(10):1093-1103. doi:10.1001/jama.2016.12195 27623463

[24] Conte MS, Farber A, Liu IH, . Open versus endovascular revascularisation for femoropopliteal disease in patients with chronic limb threatening ischaemia. Eur J Vasc Endovasc Surg. Published online February 16, 2026. doi:10.1016/j.ejvs.2026.02.018 41708051

[25] Giles KA, Farber A, Menard MT, . Surgery or endovascular therapy for patients with chronic limb-threatening ischemia requiring infrapopliteal interventions. J Vasc Surg. 2024;80(5):1515-1524. doi:10.1016/j.jvs.2024.05.049 38908805 PMC11633720

[26] Salisbury AC, Li H, Vilain KR, . Cost-effectiveness of endovascular femoropopliteal intervention using drug-coated balloons versus standard percutaneous transluminal angioplasty: results from the IN.PACT SFA II trial. JACC Cardiovasc Interv. 2016;9(22):2343-2352. doi:10.1016/j.jcin.2016.08.036 27884360

[27] Hicks CW, Holscher CM, Wang P, . Use of atherectomy during index peripheral vascular interventions. JACC Cardiovasc Interv. 2021;14(6):678-688. doi:10.1016/j.jcin.2021.01.004 33736774 PMC9069395

[28] Zafari Z, Thorlund K, FitzGerald JM, Marra CA, Sadatsafavi M. Network vs. pairwise meta-analyses: a case study of the impact of an evidence-synthesis paradigm on value of information outcomes. Pharmacoeconomics. 2014;32(10):995-1004. doi:10.1007/s40273-014-0179-1 24920194

[29] Childers CP, Lamaina M, Liu C, . *Cost-Effectiveness of Leg Bypass Versus Endovascular Therapy for Critical Limb Ischemia: A Systematic Review*. Department of Veterans Affairs; 2019. Accessed June 17, 2025. https://www.ncbi.nlm.nih.gov/books/NBK543445/31291067

[30] Adam DJ, Beard JD, Cleveland T, ; BASIL Trial Participants. Bypass versus angioplasty in severe ischaemia of the leg (BASIL): multicentre, randomised controlled trial. Lancet. 2005;366(9501):1925-1934. doi:10.1016/S0140-6736(05)67704-5 16325694

[31] Forbes JF, Adam DJ, Bell J, ; BASIL Trial Participants. Bypass Versus Angioplasty in Severe Ischaemia of the Leg (BASIL) trial: health-related quality of life outcomes, resource utilization, and cost-effectiveness analysis. J Vasc Surg. 2010;51(5 suppl):43S-51S. doi:10.1016/j.jvs.2010.01.076 20435261

[32] Moakes CA, Bradbury AW, Abdali Z, ; BASIL-2 Investigators. Vein bypass first vs. best endovascular treatment first revascularisation strategy for chronic limb-threatening ischaemia due to infra-popliteal disease: the BASIL-2 RCT. Health Technol Assess. 2024;28(65):1-72. doi:10.3310/YTFV4524 39397484 PMC11491987

[33] Cooper Z, Stiegman O, Ndumele CD, Staiger B, Skinner J. Geographical variation in health spending across the US among privately insured individuals and enrollees in Medicaid and Medicare. JAMA Netw Open. 2022;5(7):e2222138. doi:10.1001/jamanetworkopen.2022.22138 35857326 PMC9301520

[34] Duwayri YM, Aiello FA, Tracci MC, . Defining the 90-day cost structure of lower extremity revascularization for alternative payment model assessment. J Vasc Surg. 2021;73(2):662-673.e3. doi:10.1016/j.jvs.2020.06.050 32652115 PMC8189630

[35] Doshi R, Changal KH, Gupta R, . Comparison of outcomes and cost of endovascular management versus surgical bypass for the management of lower extremities peripheral arterial disease. Am J Cardiol. 2018;122(10):1790-1796. doi:10.1016/j.amjcard.2018.08.018 30217372

[36] Wang GJ, Jackson BM, Foley PJ III, . Treating peripheral artery disease in the wake of rising costs and protracted length of stay. Ann Vasc Surg. 2017;44:253-260. doi:10.1016/j.avsg.2017.01.027 28479423

[37] Levinson Z, Qureshi N, Liu JL, Whaley CM. Trends in hospital prices paid by private health plans varied substantially across the US. Health Aff (Millwood). 2022;41(4):516-522. doi:10.1377/hlthaff.2021.01476 35377759 PMC9939009

