# Supplementary material for: Cost-Effectiveness of Endovascular vs Open Surgery for Chronic Limb-Threatening Ischemia
Source: JAMA Netw Open. 2026 Aug 4;9(8):e2627335. doi: 10.1001/jamanetworkopen.2026.27335 (PMC13439427; doi:10.1001/jamanetworkopen.2026.27335)
Supplement: Supplementary file 1 [file jamanetwopen-e2627335-s001.pdf]

Supplemental Online Content

Zafari Z, Najafzadeh M, Mahesri M, et al. Cost-effectiveness of endovascular vs open surgery for chronic limb-threatening ischemia. *JAMA Netw Open*. 2026;9(8):e2627335
doi:10.1001/jamanetworkopen.2026.27335

eTable. Results of Cost-Effectiveness Analysis Presented as Incremental Costs per Clinical Event Avoided

eFigure 1. Schematic Representation of the Cost-Effectiveness Model

eFigure 2. Calibration Plots for Trial-Observed Versus Regression-Estimated Probabilities of the Clinical Outcomes (Reintervention, Amputation, MACE, and Death) Within 365 Days

eFigure 3. Trial-Observed Versus Model-Estimated Relative Risks of the Clinical Outcomes (Reintervention, Amputation, Nonfatal MI or Stroke, and Death) for Bypass Versus Endovascular Surgery

eFigure 4. Incremental Net Monetary Benefit of Endovascular Procedures With Atherectomy or Antiproliferative Drug Technology Use Versus Bypass

This supplemental material has been provided by the authors to give readers additional information about their work.

© 2026 Zafari Z et al. *JAMA Network Open*.

eTable. Results of Cost-Effectiveness Analysis Presented as Incremental Costs per Clinical Event Avoided

	Open surgery	Endovascular surgery	Difference (Endovascular minus Open)	Incremental costs per major re-intervention avoided, $ (95% CI)	Incremental costs per amputation avoided, $ (95% CI)
**5-year time horizon**					
**Cost, mean $ (95% CI)**	$118,559 ($86,978 to $157,152)	$125,535 ($96,205 to $160,357)	$6,976 (-$6,585 to $20,438)	-$52,724 (-$170,056 to $55,731)	-$197,066 (-$1,903,460 to $1,405,687)
**Rate of major re-intervention**	0.098 (0.077, 0.121)	0.236 (0.175, 0.306)	0.137 (0.072, 0.210)		
**Rate of above ankle amputation**	0.115 (0.092, 0.138)	0.151 (0.104, 0.206)	0.036 (-0.015, 0.096)		
**10-year time horizon**					
**Cost, mean $ (95% CI)**	$179,074 ($134,547 to $232,757)	$186,598 ($145,023 to $234,422)	$7,523 (-$14,052 to $28,854)	-$42,999 (-$175,920 to $92,515)	-$164,752 (-$1,771,824 to $1,298,878)
**Rate of major re-intervention**	0.153 (0.128 to 0.180)	0.330 (0.254 to 0.414)	0.177 (0.094 to 0.265)		
**Rate of above ankle amputation**	0.187 (0.159 to 0.215)	0.236 (0.172 to 0.308)	0.049 (-0.021 to 0.128)		

© 2026 Zafari Z et al. *JAMA Network Open*.

eFigure 1. Schematic Representation of the Cost-Effectiveness Model

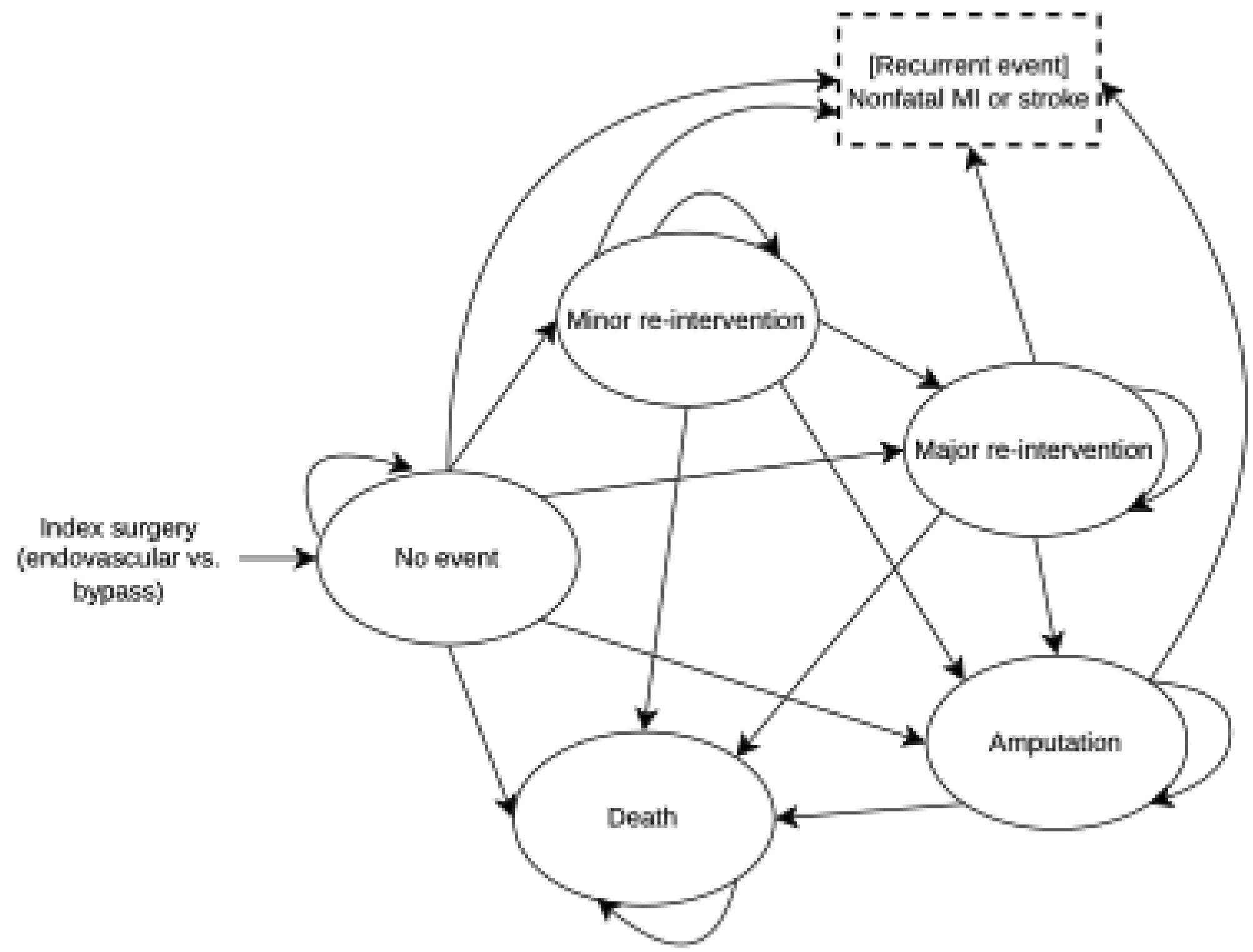

MI: myocardial infarction.

© 2026 Zafari Z et al. *JAMA Network Open*.

eFigure 2. Calibration Plots for Trial-Observed Versus Regression-Estimated Probabilities of the Clinical Outcomes (Reintervention, Amputation, MACE, and Death) Within 365 Days

(A) Calibration plot for re-intervention

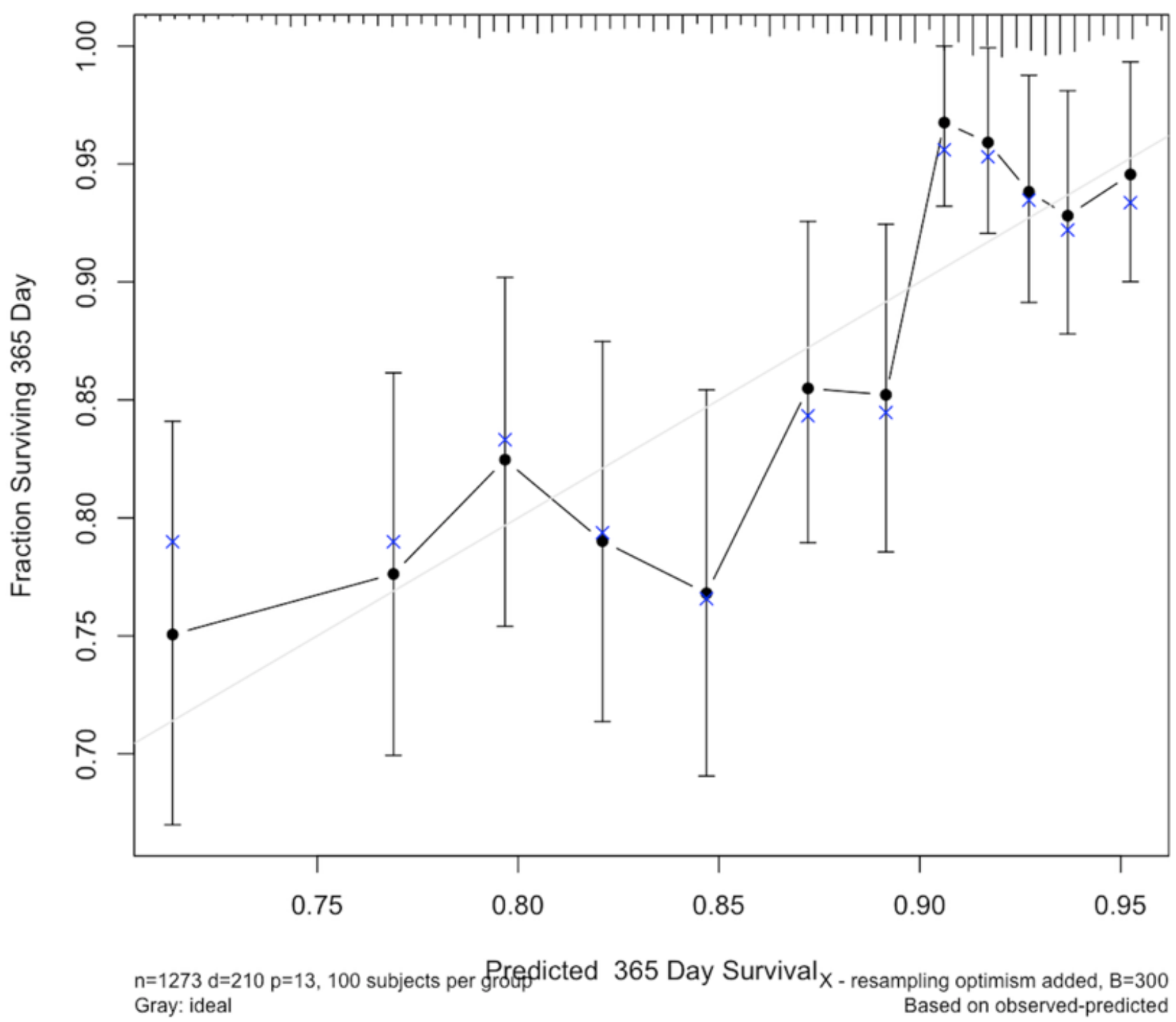

© 2026 Zafari Z et al. *JAMA Network Open*.

(B) Calibration plot for amputation

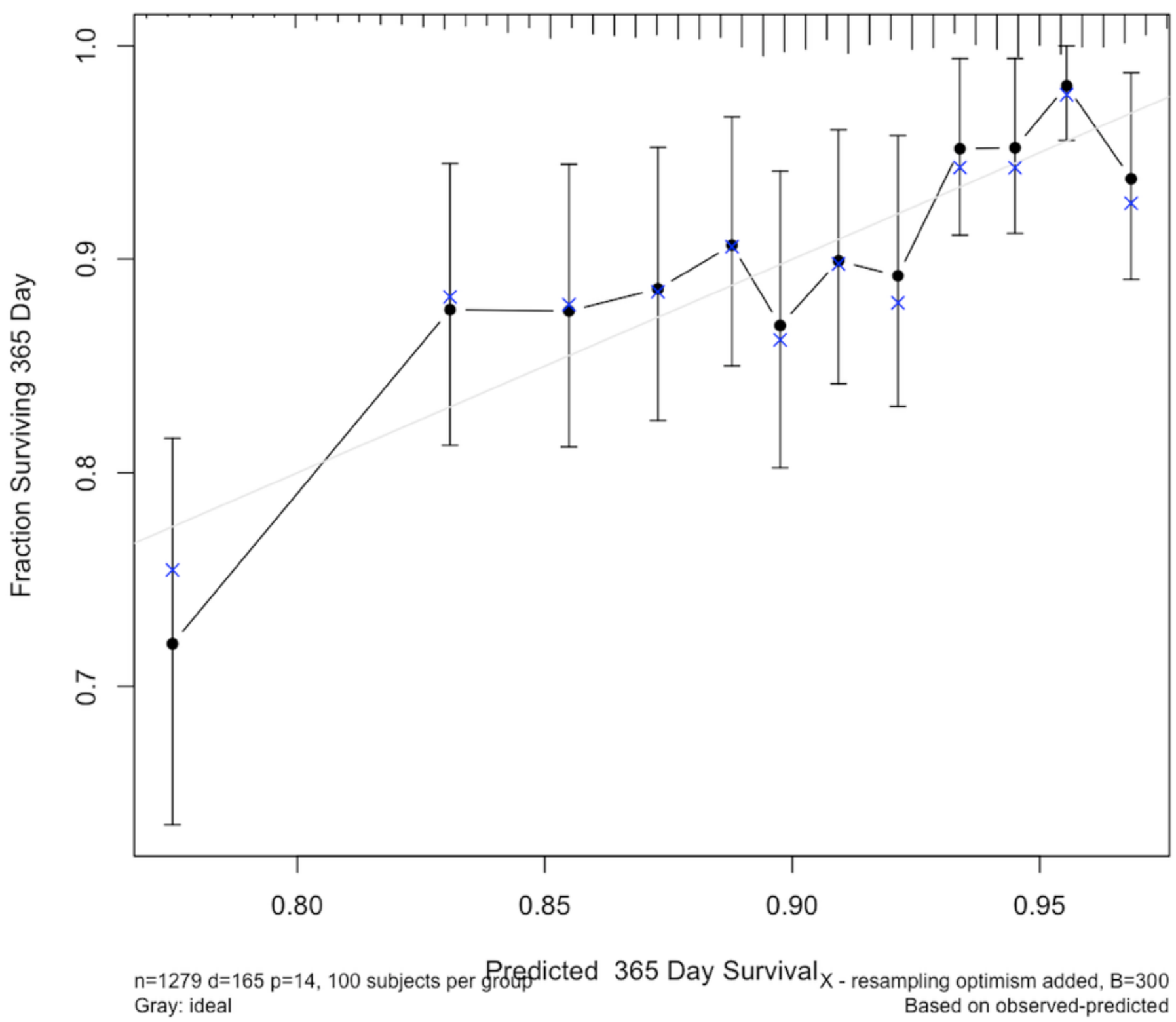

© 2026 Zafari Z et al. *JAMA Network Open*.

(C) Calibration plot for major adverse cardiovascular events

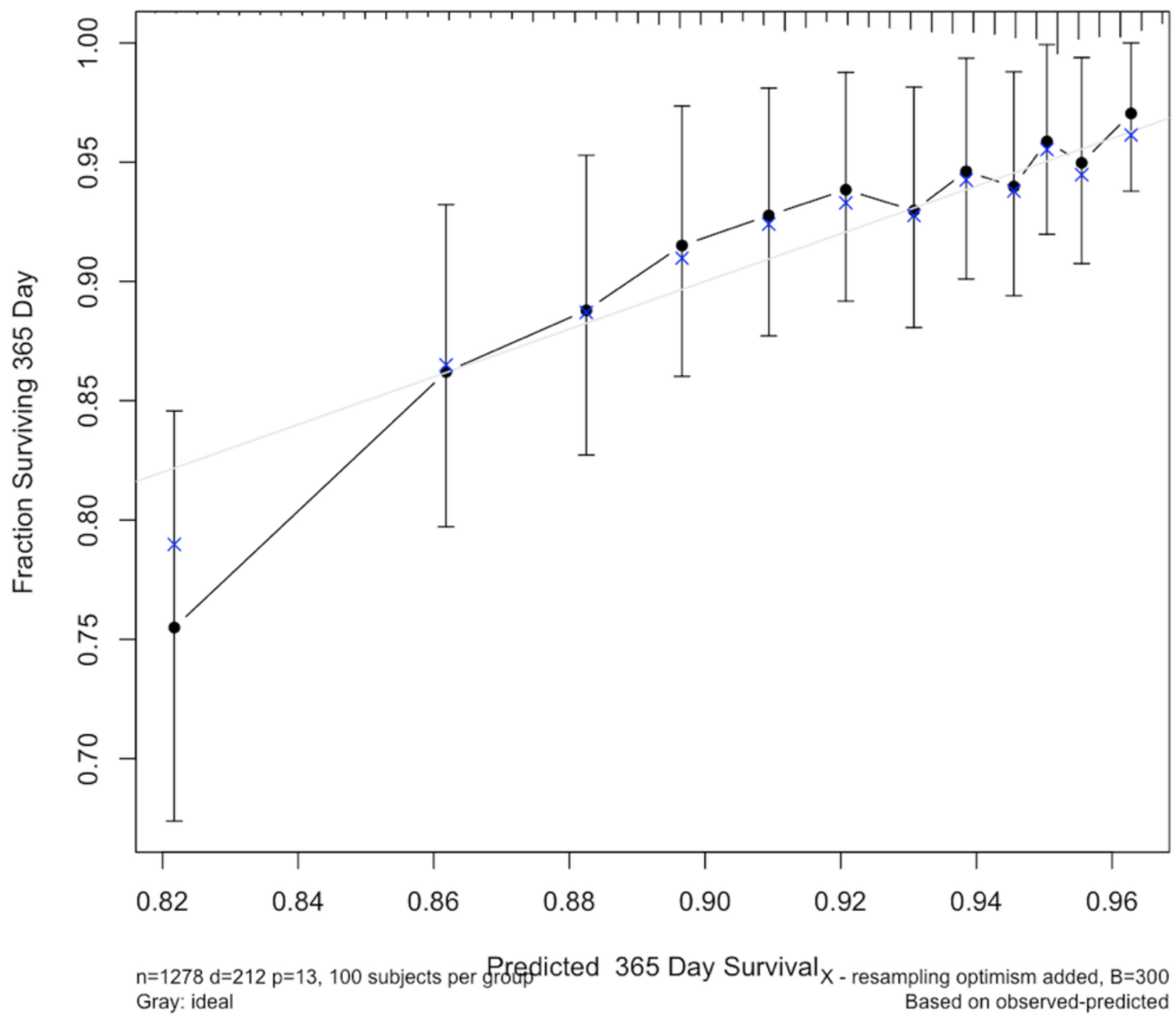

© 2026 Zafari Z et al. *JAMA Network Open*.

(D) Calibration plot for death

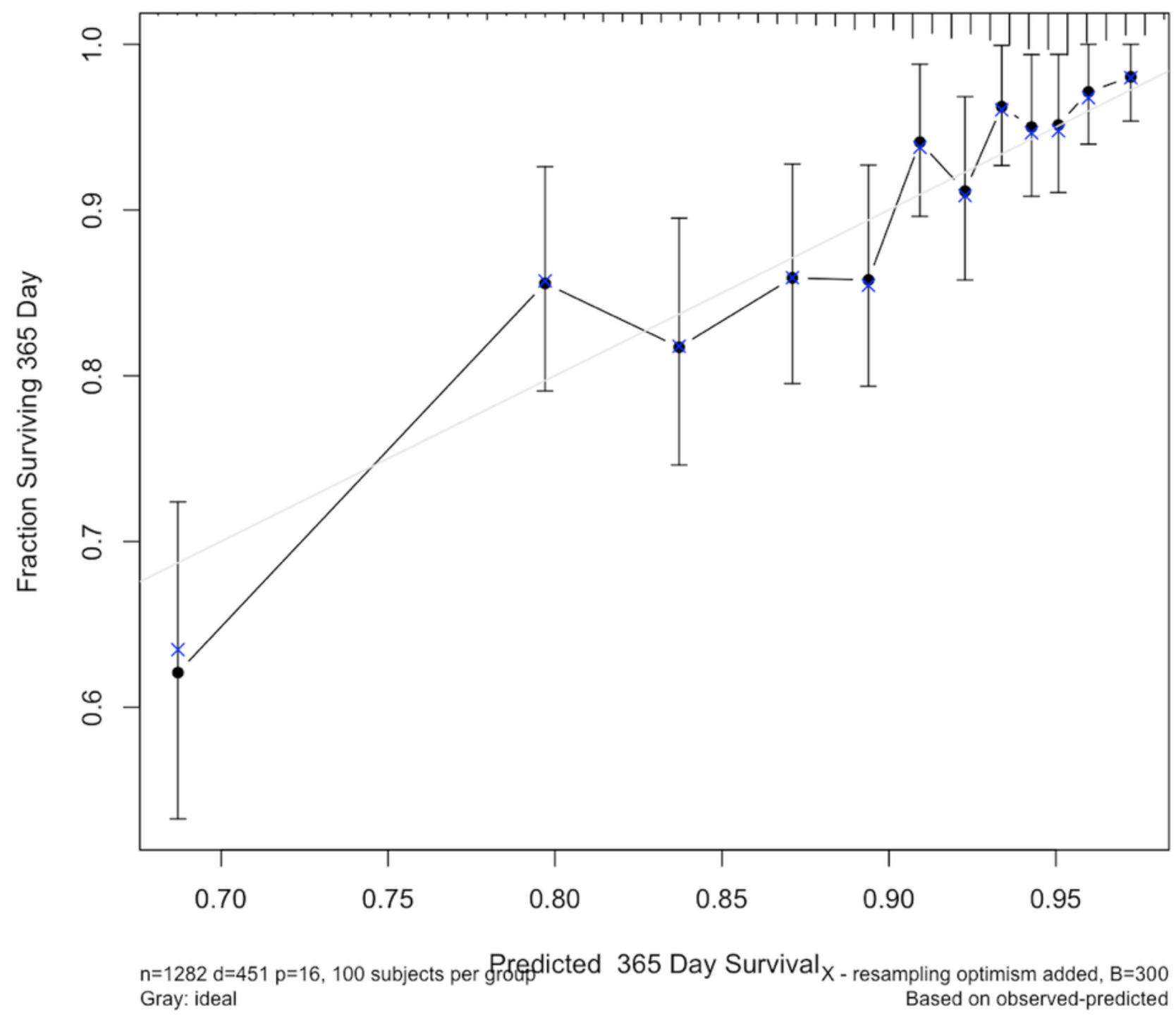

© 2026 Zafari Z et al. *JAMA Network Open*.

eFigure 3. Trial-Observed Versus Model-Estimated Relative Risks of the Clinical Outcomes (Reintervention, Amputation, Nonfatal MI or Stroke, and Death) for Bypass Versus Endovascular Surgery

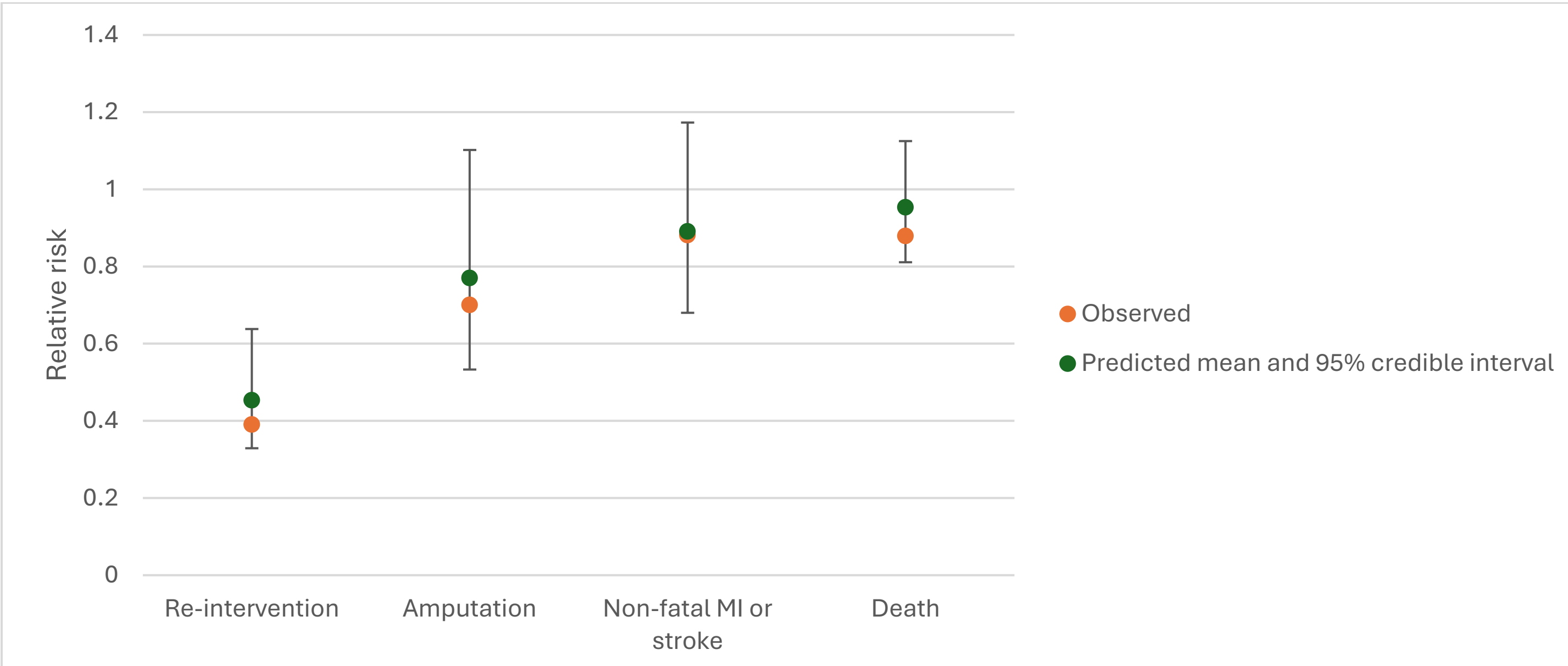

MI: myocardial infarction.

© 2026 Zafari Z et al. *JAMA Network Open*.

eFigure 4. Incremental net monetary benefit of endovascular procedures with atherectomy or antiproliferative drug technology use versus Bypass.

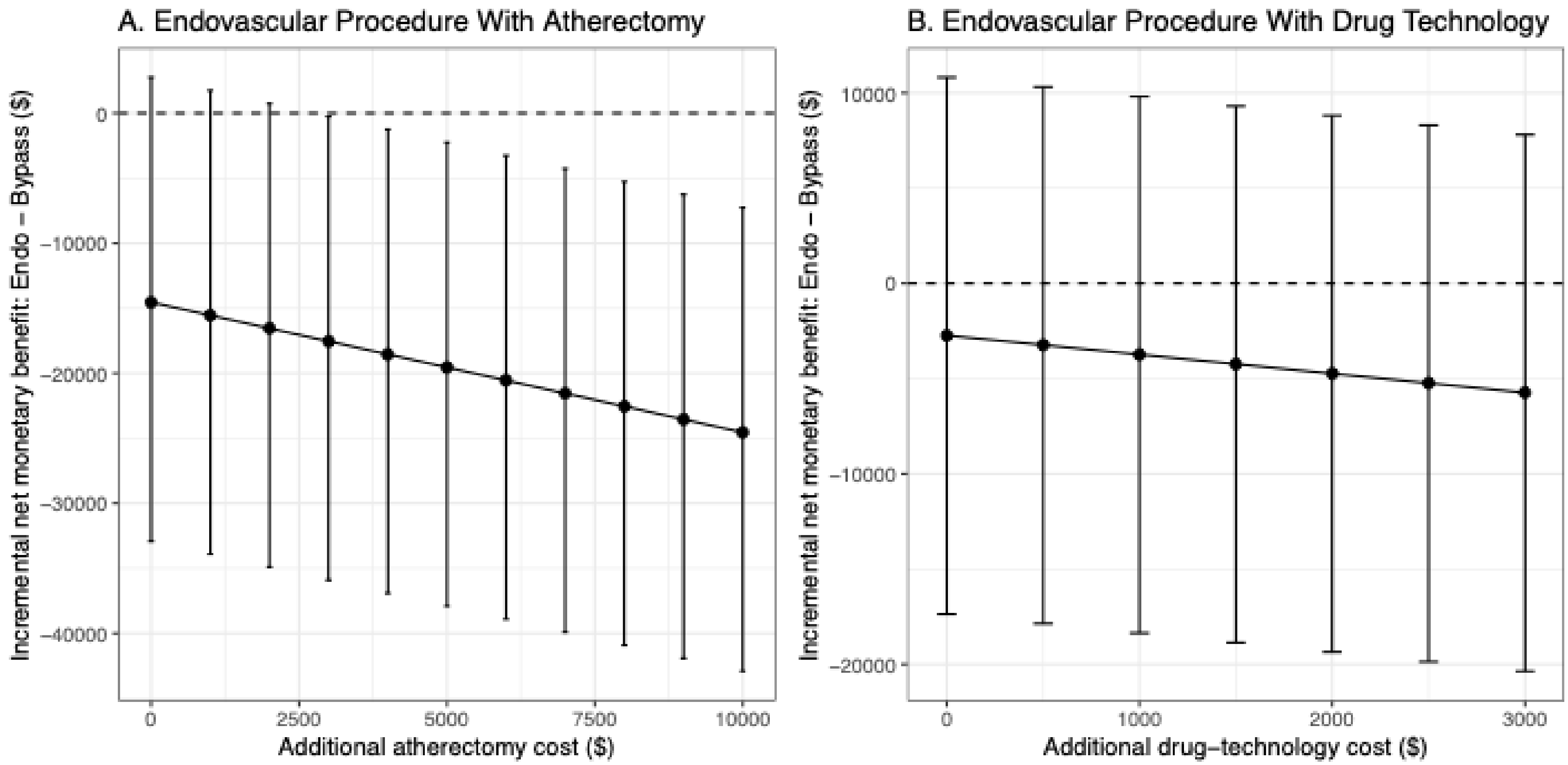

© 2026 Zafari Z et al. *JAMA Network Open*.
