# Supplementary material for: Cost-Effectiveness of Endovascular vs Open Surgery for Chronic Limb-Threatening Ischemia
Source: JAMA Netw Open. 2026 Aug 4;9(8):e2627335. doi: 10.1001/jamanetworkopen.2026.27335 (PMC13439427; doi:10.1001/jamanetworkopen.2026.27335)
Supplement: Supplementary file 2 [file jamanetwopen-e2627335-s002.pdf]

Data Sharing Statement

Data
Data available: No
